# Cells responsible for liver mass regeneration in rats with 2-acetylaminofluorene/partial hepatectomy injury

**DOI:** 10.1186/s12929-018-0441-5

**Published:** 2018-04-25

**Authors:** Chin-Sung Chien, Ya-Hui Chen, Hui-Ling Chen, Chiu-Ping Wang, Shang-Hsin Wu, Shu-Li Ho, Wen-Cheng Huang, Chun-Hsien Yu, Mei-Hwei Chang

**Affiliations:** 1 Department of Pediatrics, Taipei Tzu Chi Hospital, Buddhist Tzu Chi Medical Foundation. No.289, Jianguo Rd., Xindian Dist, New Taipei City, 23142 Taiwan; 2 Graduate Institute of Clinical Medicine, College of Medicine, National Taiwan University . No.7, Chung Shan South Rd., Zhongzheng Dist, Taipei, 10002 Taiwan; 3 Hepatitis Research Center, National Taiwan University Hospital. No.1, Changde St., Zhongzheng Dist, Taipei, 10048 Taiwan; 4 Department of Pediatrics, School of Medicine, Tzu Chi University, No.701, Sec. 3, Zhongyang Rd, Hualien, 97004 Taiwan; 5 Department of Pediatrics, National Taiwan University Hospital, and College of Medicine, National Taiwan University. No.8, Chung Shan South Rd., Zhongzheng Dist, Taipei, 10041 Taiwan

**Keywords:** AAF/PH, Ductular reaction, Hepatocyte, Liver injury, Oval cells, Rat, Regeneration

## Abstract

**Background:**

Whether hepatic progenitor cells (HPCs)/oval cells regenerate liver mass upon chronic liver injury is controversial in mice and has not been conclusively proven in humans and rats. In this study, we examined which cell type—hepatocytes or oval cells—mediates liver regeneration in the classic rat 2-acetylaminofluorene (AAF)/partial hepatectomy (PH) injury where AAF reversibly blocks hepatocyte proliferation, thereby inducing oval cell expansion after the regenerative stimulus of PH.

**Methods:**

We employed lineage tracing of dipeptidyl peptidase IV (DPPIV, a hepatocyte canalicular enzyme)-positive hepatocytes by subjecting rats with DPPIV-chimeric livers to AAF/PH, AAF/PH/AAF (continuous AAF after AAF/PH to nonselectively inhibit regenerating hepatocytes), or AAF/PH/retrorsine injury (2-dose retrorsine after AAF/PH to specifically and irreversibly block existing hepatocytes); through these methods, we determined hepatocyte contribution to liver regeneration. To determine the oval cell contribution to hepatocyte regeneration, we performed DPPIV(+) oval cell transplantation combined with AAF/PH injury or AAF/PH/retrorsine injury in DPPIV-deficient rats to track the fate of DPPIV(+) oval cells.

**Results:**

DPPIV-chimeric livers demonstrated typical oval cell activation upon AAF/PH injury. After cessation of AAF, DPPIV(+) hepatocytes underwent extensive proliferation to regenerate the liver mass, whereas oval cells underwent hepatocyte differentiation. Upon AAF/PH/AAF injury where hepatocyte proliferation was inhibited by continuous AAF treatment following AAF/PH, oval cells extensively expanded in an undifferentiated state but did not produce hepatocytes. By substituting retrorsine for AAF administration following AAF/PH (AAF/PH/retrorsine), oval cells regenerated large-scale hepatocytes.

**Conclusions:**

Hepatocyte self-replication provides the majority of hepatocyte regeneration, with supplementary contribution from oval cells in rats under AAF/PH injury. Oval cells expand and maintain in an undifferentiated state upon continuously nonselective liver injury, whereas they can significantly regenerate hepatocytes in a noncompetitive environment.

## Background

Chronic liver disease is a major health concern worldwide. Although the liver has notable regenerative capacity, the nature of impaired regeneration resulting from chronic liver damage remains unclear [1–3]. Understanding how a diseased liver is repaired may require identification and knowledge of the cellular mechanisms that govern liver regeneration; such knowledge would aid in the development of new therapeutic strategies. Severe or chronic liver injury is often associated with hepatocyte replicative senescence. This leads to the activation of hepatic progenitor cells (HPCs), the human equivalent of oval cells in rodents [1–3]. However, the relative contribution of residual hepatocytes and activated HPCs/oval cells to parenchymal regeneration remains controversial [3–6].

Oval cells in rats were revealed in studies where the combination of chemical DNA damage by 2-acetylaminofluorene (AAF) to block hepatocyte proliferation and partial hepatectomy (PH) to stimulate regeneration provoked the extensive emergence of oval cells from the biliary tree [7, 8]. Thymidine-labeling studies have suggested that these oval cells could become hepatocytes and replace liver mass [8, 9]. The suggestion that oval cells regenerate liver mass in rats is consistent with clinical observations in cases of human chronic liver disease, where hepatocytes became senescent and HPCs could give rise to regenerative hepatocyte nodules [10–12]. However, the hypothesis that oval cells produce hepatocytes upon liver injury has recently been challenged. Studies employing sophisticated lineage tracing tools in mice and zebrafish have yielded conflicting results [13–24]. Some studies have revealed that oval cells contribute significantly to hepatocyte regeneration [13–17], whereas other studies have reported little to no contribution [18–24]. These studies differed widely in the severity of liver injury. In the studies in which oval cells made little to no contribution, the liver injuries were not sufficiently severe: the remaining hepatocytes could undergo proliferation; by contrast, hepatocytes were completely inhibited or extremely ablated in the studies in which significant contributions were reported. That hepatocytes are senescent in human chronic liver disease or incapable of proliferating in rats following AAF/PH injury is well known; however, whether HPCs/oval cells can produce hepatocyte nodules in humans or regenerate liver mass in rats not conclusively proven, thus necessitating lineage-tracing studies [3, 6].

In the present study, we aimed to determine which cell type—hepatocytes or oval cells—mediates the regeneration of liver mass in the classic rat AAF/PH model. To this end, we used rats with dipeptidyl peptidase IV (DPPIV, a hepatocyte canalicular enzyme)-chimeric livers, which were composed of DPPIV(+) and DPPIV(−) hepatocytes and uniformly DPPIV(−) biliary epithelial cells (BECs) [25–27]. DPPIV(−) hepatocytes are incapable of proliferation. Rats with DPPIV-chimeric livers have been used to track the role of hepatocytes in organ regeneration [25–27]. We subjected the rats to AAF/PH injury to examine the hepatocyte contribution through lineage tracing of the DPPIV(+) hepatocytes. To determine the oval cell contribution, we performed transplantation of DPPIV(+) oval cells into DPPIV(−) rat livers and subjected the rats to AAF/PH injury. The experiments revealed that hepatocytes are the primary cells involved in the regeneration of liver mass in rats subjected to AAF/PH injury and that oval cells can differentiate into hepatocytes, contributing supplementarily to liver regeneration. Given these results, to explore whether oval cells can regenerate liver mass given an appropriate environment, we experimented with two additional types of liver injury: extending AAF administration after the AAF/PH protocol to nonselectively inhibit regenerating hepatocytes (AAF/PH/AAF injury) and administering two doses of retrorsine following AAF/PH injury to specifically and irreversibly block existing hepatocytes (AAF/PH/retrorsine injury).

## Methods

### Animals [28]

Male DPPIV-deficient F344 rats, provided by Professor Gupta of the Albert Einstein College of Medicine, were used as recipient animals. Male wild-type F344 rats were purchased from the National Laboratory Animal Center, Taiwan, and used as donor animals. All the animals received humane care in compliance with the guidelines of the National Science Council of Taiwan (NSC, 1997). All animal experiments were approved by the Institutional Animal Care and Use Committee, Taipei Tzu Chi Hospital, Buddhist Tzu Chi Medical Foundation, and the Institutional Laboratory Animal Care and Use Committee of National Taiwan University, College of Medicine and College of Public Health.

### Generation of rats with DPPIV-chimeric livers

The rats with DPPIV-chimeric livers were generated according to the method employed in our previous studies [26–28]. In brief, male DPPIV-deficient rats received two doses of retrorsine (30 mg/kg, i.p.; Sigma, St. Louis, MO, USA) to permanently block DPPIV(−) hepatocyte proliferation and D-galactosamine (0.7 g/kg, i.p.; Sigma) to create a regenerative stimulus. The rats then received DPPIV(+) hepatocyte transplantation (1 × 10^7^/mL) intraportally 24 h after D-galactosamine treatment. The rats were allowed to recover for 1 or 2 months. The degree of colonization by DPPIV(+) hepatocytes constructed through this protocol was approximately 50% at 1 month and 40% at 2 and 3 months [26–28]. DPPIV-chimeric livers examined at 1 month after DPPIV(+) hepatocyte transplantation revealed essentially normal histology. DPPIV(+) hepatocytes were completely integrated into the hepatic plates and were histologically identical to the surrounding hepatocytes [26].

### Isolation and characterization of oval cells

Oval cells were isolated as outlined by Yovchev et al. and in our earlier papers [27, 29]. In brief, in situ liver perfusion, collagenase digestion, and differential centrifugation were performed to purify the oval cells from D-galactosamine treated wild-type male F344 rats at 5 days after D-galactosamine treatment, the time at which the livers accumulate the highest number of oval cells in this model. Each preparation had > 95% viable cells, as assessed by trypan blue exclusion. The abundance of oval cells in each preparation were identified through CK-19 and gamma-glutamyl-transpeptidase (GGT) staining [27–29].

### AAF/PH experiments

#### AAF/PH experiment

AAF (Sigma, St. Louis, MO) 4 mg/mL suspended in 1% dimethylcellulose (8 mg/kg daily) was given to the rats with DPPIV-chimeric livers over 6 consecutive days through gavage. One-third PH was performed on day 7, which was followed by six additional AAF treatments. The rats were then returned to the basal diet until the indicated sacrifice time points at 1, 2, and 4 weeks following cessation of AAF.

#### AAF/PH/AAF experiment

The group was first treated with the AAF/PH protocol as described earlier in the text and then by continuous AAF gavage daily for an additional 4 weeks. Rats were sacrificed at the indicated time points.

#### AAF/PH/retrorsine experiment

The group was first treated with the AAF/PH protocol and then returned to the basal diets. The rats then received two doses of retrorsine (30 mg/kg, i.p.) 2 weeks apart, at 1 and 3 weeks after cessation of AAF. They were sacrificed at the indicated time points.

### Histochemical and immunohistochemical analysis

Histological analysis was conducted using 6-μm frozen liver sections. DPPIV expression was detected through enzyme histochemical staining as previously described [30, 31]. GGT was determined based on the method of Rutenberg et al. [32, 33]. Dual immunofluorescence staining for OV6 (R&D Systems, Minneapolis, MN, USA; 1:600) and DPPIV (R&D; 1:100); Ki67 (BD pharmingen™, BD Biosciences, NJ, USA; 1:100) and DPPIV; CK19 (Novocastra, Newcastle, UK; 1:100) and DPPIV; C/EBP*α* (Santa Cruz Biotechnology, Santa Cruz, CA, USA; 1:200) and DPPIV; hepatocyte nuclear factor-4α (HNF4α) (Santa Cruz Biotechnology; 1:50) and DPPIV; laminin (DAKO, CA, USA; 1:1000) and DPPIV; CK19 and C/EBP*α*; CK19 and carbamoyl-phosphate-synthetase 1 (CPS1) (Santa Cruz Biotechnology; 1:100); OV6 and laminin; OV6 and HNF4α; OV6 and C/EBP*α*; EpCAM (US Biological, Swampscott, MA, USA; 1:100) and HNF4α; Ki67 and pan-cytokeratin (pan-CK) (DAKO; 1:600); and Ki67 and EpCAM were detected using the method described by Paku et al. [34]. Secondary antibodies used in the experiments included Alexa Fluor 488 donkey anti-mouse IgG (Molecular Probes, Oregon, USA; 1:200), Alexa Fluor 594 donkey anti-goat IgG (Molecular Probes; 1:200), and Alexa Fluor 594 donkey anti-rabbit IgG (Molecular Probes; 1:200). Nuclei were labeled using 4′,6-diamidino-2-phenylindole (Molecular Probes).

### Morphometrical analysis

Three or four sections from multiple liver lobes per rat were stained for DPPIV activity to determine the area occupied by DPPIV(+) hepatocytes. To ensure coverage of the entire section, microphotographs were captured from consecutively adjacent areas under 100× magnification by using a digital camera. The area was quantitated using ImageJ software (National Cancer Institute, Bethesda, MD, USA) [28].

### Proliferation index

The proliferation index (**PI**, expressed as a percentage [28]) was determined in both DPPIV(+) and pan-CK(+) cells in 10 microphotographs randomly selected from the microscopic fields of each rat. The numbers of Ki67-stained nuclei were counted under 100× magnification.

### Statistical analysis

Data were presented as means ± SD. The significance of differences was analyzed using a t-test or ANOVA with the LSD method as appropriate using SPSS 22.0 (Chicago, IL, USA). *P* < 0.05 was considered statistically significant.

## Results

### AAF/PH treatment activates typical oval cell response in rats with DPPIV-chimeric livers

**Fig. 1:**
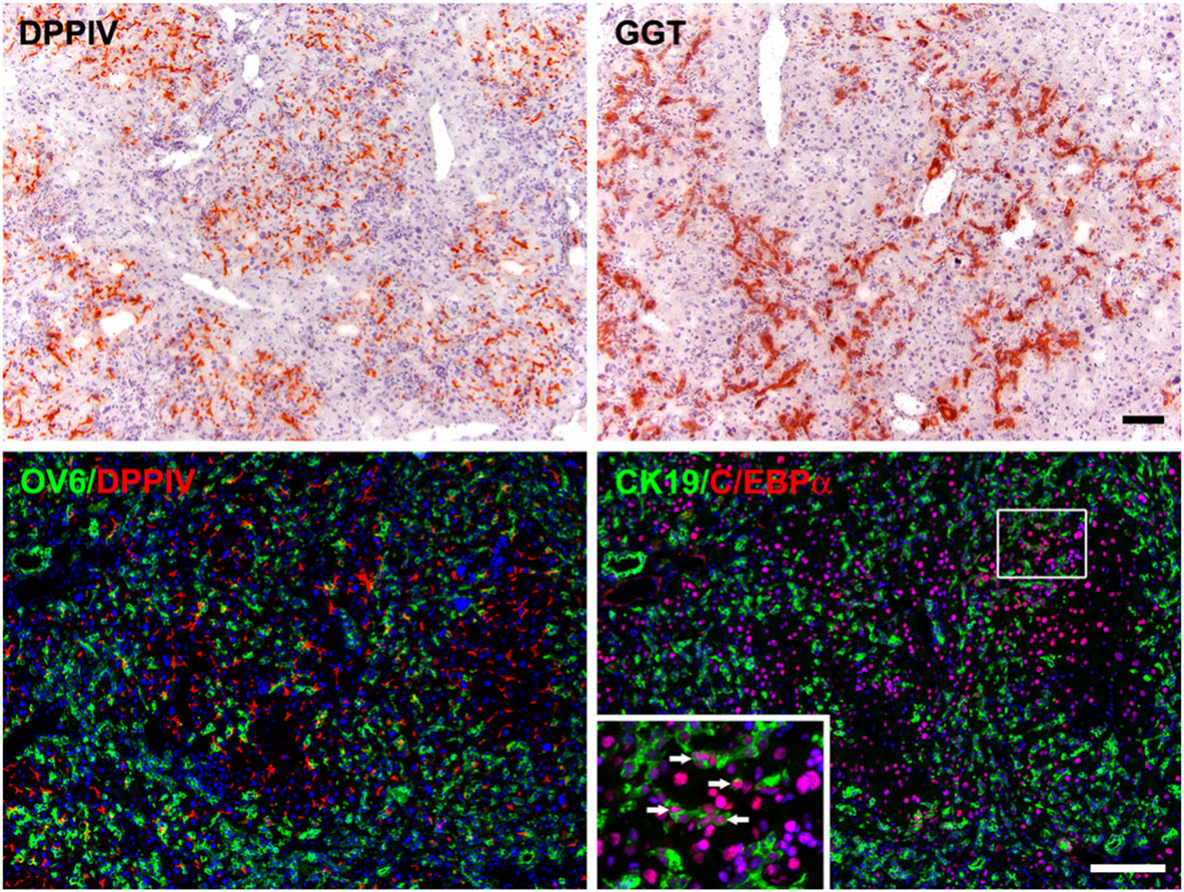
DPPIV-chimeric livers demonstrate typical oval cell response upon AAF/PH injury. DPPIV-chimeric liver sections 1 week after AAF/PH injury were analyzed using histochemical staining for DPPIV (red) and gamma-glutamyl-transpeptidase (GGT; red) and dual immunofluorescence staining for OV6 (green)/DPPIV (red) and CK19 (Green)/C/EBPα (red) (arrows). Marked oval cell expansion was observed. Original magnification: 100×/zoom magnification 200×. Scale bars: 100 μm

DPPIV-chimeric livers are characterized by the presence of both DPPIV(+) and DPPIV(−) hepatocytes along with uniformly DPPIV(−) BECs [25–27]. The DPPIV(−) hepatocytes are incapable of proliferation. We conducted pilot studies by subjecting rats with DPPIV-chimeric livers to classic AAF/two-thirds PH protocol. This protocol results in high mortality rates in rats. Therefore, we modified the protocol to AAF/one-third PH and confirmed that a typical oval cell response was present in the DPPIV-chimeric livers 1 week after AAF/PH injury, which was identified by the robust expansion of DPPIV(−)/GGT(+) (a marker of fetal hepatoblasts and oval cells) [33] cell cords from the periportal regions into the parenchyma (Fig. 1). These oval cells were stained positive for OV6 (an oval cell marker) and CK19 (a biliary cell marker). Some oval cells expressed C/EBPα (an early gene of hepatocyte lineage) [35].

### Hepatocytes are the primary cells responsible for regeneration of liver mass in AAF/PH injury

**Fig. 2:**
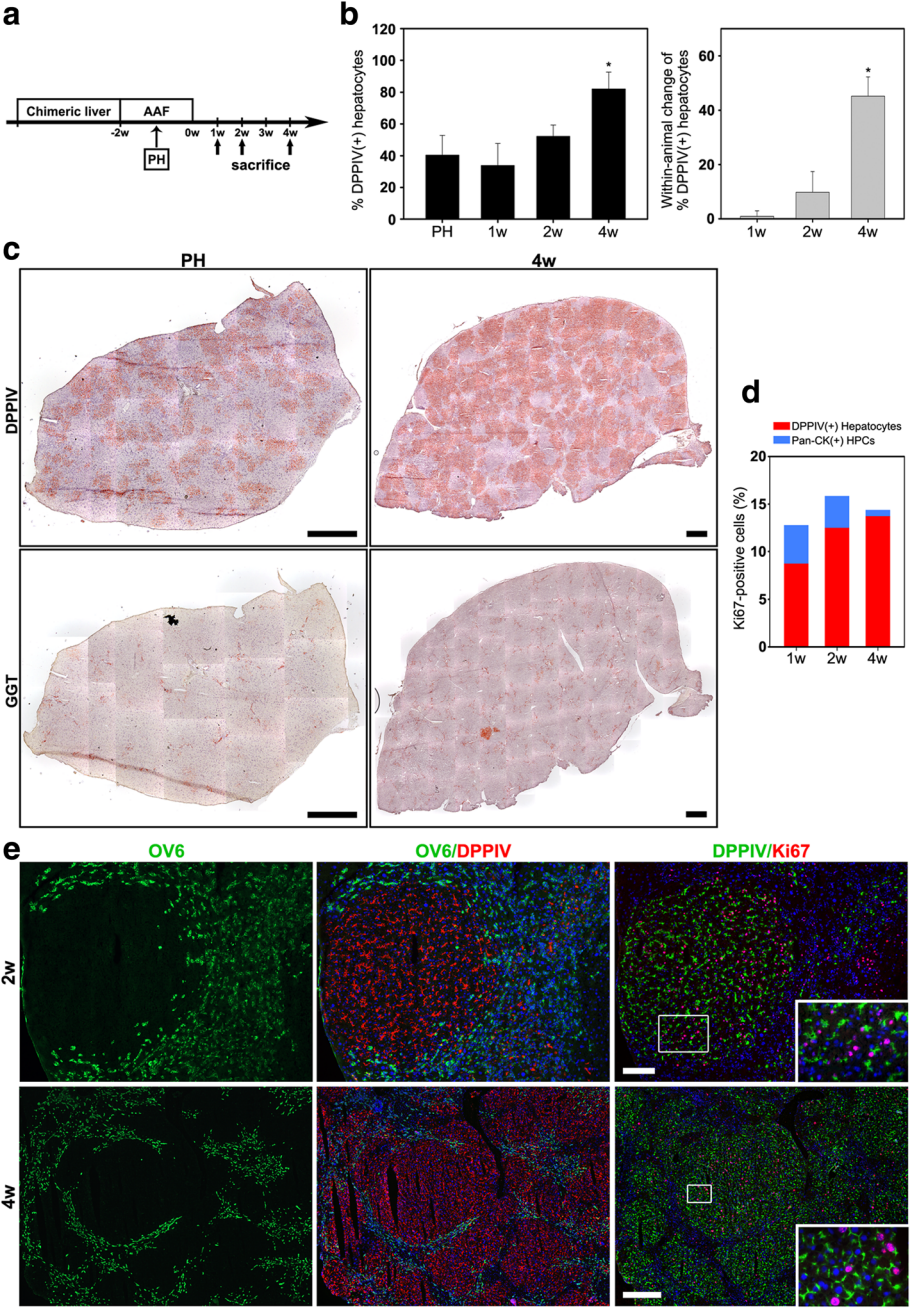
Hepatocytes were the primary cells responsible for the regeneration of liver mass in the AAF/PH injury model. **a** Scheme illustrating DPPIV-chimeric lineage tracing system subjected to AAF/PH treatment. **b** Mean proportions of DPPIV(+) hepatocytes in the DPPIV-chimeric liver at the time of PH, 1, 2, and 4 weeks after AAF/PH injury. Within-animal comparison indicated that recovery from AAF/PH injury was associated with a marked increase in DPPIV(+) hepatocytes. * *P* < 0.05, 4 weeks vs. PH, ANOVA with LSD. Each bar represents the mean ± SD of six rats. **c** Serial liver sections stained histochemically for DPPIV and gamma-glutamyl- transpeptidase (GGT, a marker of oval cells) at the time of PH and 4 weeks after AAF/PH injury. **d** Quantification of proliferative activities of DPPIV(+) hepatocytes and oval cells at 1, 2, and 4 weeks after AAF/PH injury. **e** Representative double-immunofluorescence images for DPPIV/OV6 and DPPIV/Ki67 in serial liver sections at 2 and 4 weeks after AAF/PH injury demonstrate proliferating DPPIV(+) hepatocyte clusters. Original magnification: **c** 100×; **e** 2 weeks, 100×; **e** 4 weeks, 40×/zoom magnification 400×. Scale bars: **c** 300 μm; **e** 2 weeks, 100 μm; **e** 4 weeks, 300 μm

We subjected rats with DPPIV-chimeric livers to modified AAF/PH treatment; harvested the livers at 1, 2, and 4 weeks after AAF termination (Fig. 2a); and determined the percentage of DPPIV(+) hepatocytes in the livers through hepatectomy at the indicated time points. The rationale was that if DPPIV(−) oval cells were the driving force of parenchymal regeneration, expansion of oval cell–derived hepatocytes would have caused a reduction in the area percentage of DPPIV(+) hepatocytes. Alternatively, if new hepatocytes were derived from existing hepatocytes, the area percentage of DPPIV(+) hepatocytes would have increased [19, 22].

The liver/body weight ratios at 1, 2, and 4 weeks after AAF/PH injury were 2.6% ± 0.4%, 3.0% ± 0.5%, and 3.2% ± 0.2%, respectively (*P* < 0.05, 4 weeks vs. 1 week, ANOVA with LSD), indicating regeneration of liver mass with time. The mean areas of DPPIV(+) hepatocytes in the DPPIV-chimeric liver at the time of PH and 1, 2, and 4 weeks after AAF/PH injury were 40.4% ± 12.4%, 33.9% ± 13.9%, 52.2% ± 7.2%, and 82.2% ± 10.3%, respectively (*P* < 0.05, 4 weeks vs. PH, ANOVA with LSD) (Fig. 2b). The average increase in area percentage of DPPIV(+) hepatocytes after a 4-week recovery within animal was 40.9% ± 15.1% (*n* = 6; *P* < 0.01, 4 weeks vs. PH, t-test), indicating that regeneration of liver mass after AAF/PH injury is associated with a marked increase in DPPIV(+) hepatocytes (Fig. 2b and c). At 4 weeks, ductular oval cells were decreased*.* GGT(+)/DPPIV(−) foci were rarely found (Fig. 2c).

To ascertain whether DPPIV(+) hepatocytes were responsible for the regeneration of liver mass, we conducted double-immunofluorescence staining for DPPIV/CK19, DPPIV/Ki67, and pan-CK/Ki67 in serial sections to determine the proliferative index of DPPIV(+) hepatocytes and oval cells. Ki67 expression was observed in both DPPIV(+) hepatocytes and DPPIV(−) oval cells at each time point. The proliferative index of DPPIV(+) hepatocytes was 2.2-, 3.7-, and 20.7-fold higher than that of oval cells at 1, 2, and 4 weeks respectively (Fig. 2d and e). These results further evidence that hepatocytes are the primary cells responsible for the regeneration of liver mass following AAF/PH injury.

### Oval cells can give rise to hepatocytes and provide a supplementary contribution to hepatocyte regeneration in AAF/PH injury

**Fig. 3:**
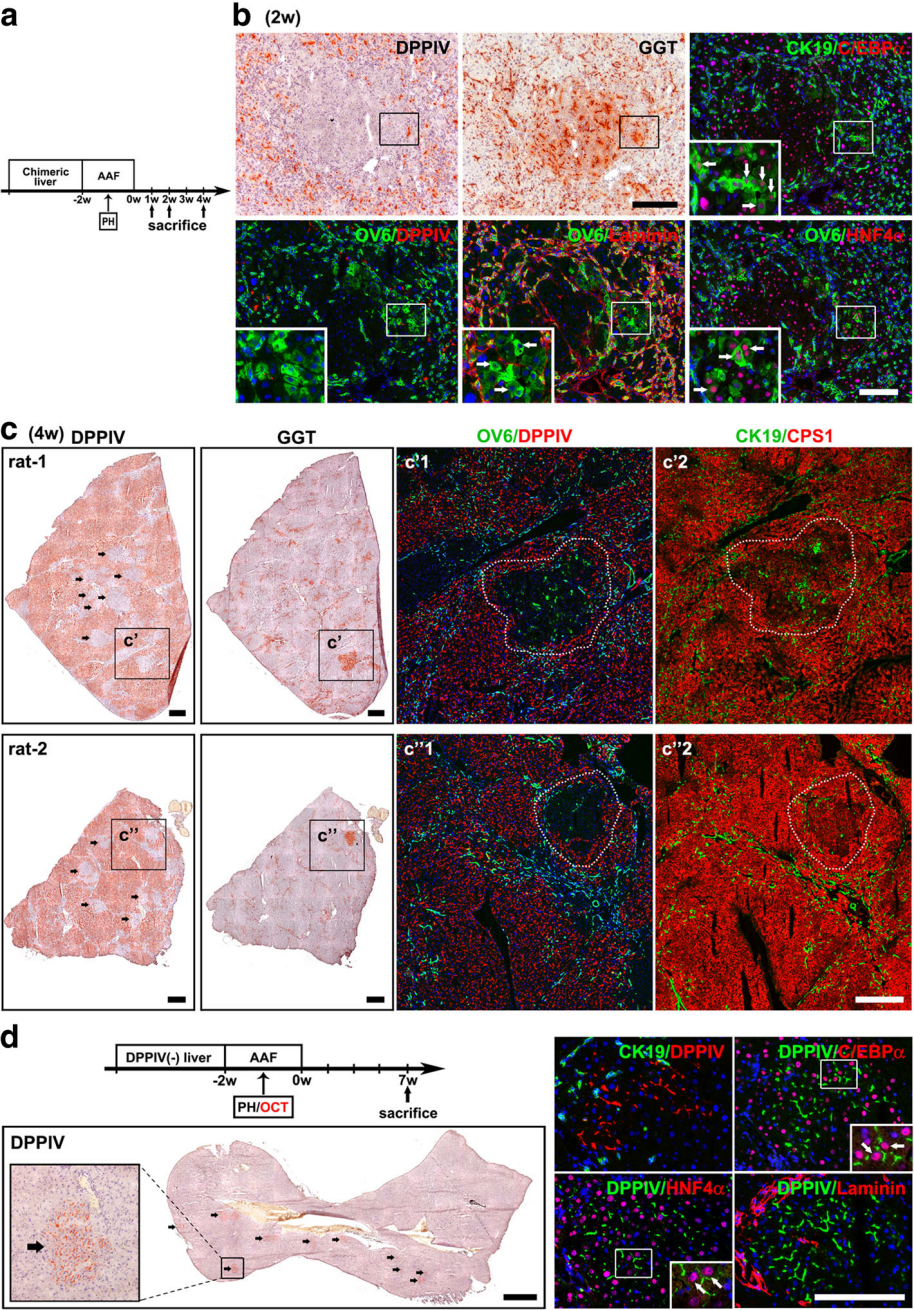
Oval cells give rise to hepatocytes after AAF/PH injury but are not the primary contributor to hepatocyte regeneration. **a** Scheme illustrating DPPIV-chimeric lineage tracing system subjected to AAF/PH treatment. Representative histochemical and double-immunofluorescence images in serial liver sections at (**b**) 2 weeks and (**c**) 4 weeks after AAF/PH injury. **b** GGT(+)/DPPIV(−) foci are composed of hepatocytes [OV6(−)/HNF4α(+), CK19(−)/C/EBPα(+), CK19(−)/CPS1(+)] and differentiating oval cells [rectangle areas; OV6(+)/HNF4α(+), CK19(+)/C/EBPα(+), OV6(+)/Laminin(−)], which were in connection with the oval cell proliferation [OV6(+)/HNF4α(−), CK19(+)/C/EBPα(−)]. **c** Whole liver sections of DPPIV-chimeric livers from different rats at 4 weeks after AAF/PH injury demonstrate the contribution of oval cell–derived DPPIV(−) hepatocytes to liver regeneration after AAF/PH injury. **d** DPPIV-deficient rats received DPPIV(+) oval cells transplantation combined with AAF/PH injury. After 7 weeks following AAF/PH injury, DPPIV(+) oval cells regenerated DPPIV(+) hepatocyte clusters (arrows). At higher magnification, DPPIV(+) oval cell–derived hepatocytes were histologically identical to the surrounding DPPIV(−) hepatocytes. Dual immunofluorescence staining showed that DPPIV(+) oval cell–derived hepatocytes expressed DPPIV(+)/HNF4α(+) and DDPPIV(+)/C/EBPα(+). Original magnification: **b** 100×/zoom magnification 200×; **c** histochemical 100×/ double-immunofluorescence 40×; **d** histochemical 100×/zoom magnification 200×/ double-immunofluorescence 100×/zoom magnification 400×. Scale bars: **b** 100 μm; **c** 300 μm; **d** histochemical 300 μm/ double-immunofluorescence 100 μm

Liver sections at 1, 2, and 4 weeks after AAF termination were examined for evidence of oval-cell-to-hepatocyte differentiation (Fig. 3a). We observed numerous GGT(+)/DDPIV(−) foci adjacent to the oval cell proliferation at 2 and 4 weeks. Dual immunofluorescence staining in serial sections revealed that these foci were composed of differentiated hepatocytes [OV6(−)/HNF4α(+), CK19(−)/C/EBPα(+), CK19(−)/CPS1(+) (hepatocyte specific enzyme)] and differentiating hepatic oval cells [OV6(+)/HNF4α(+), CK19(+)/C/EBPα(+), OV6(+)/Laminin(−)] which were in connection with the oval cells proliferation [OV6(+)/HNF4α(−), CK19(+)/C/EBPα(−)] (Fig. 3b and c). This finding suggests that oval cells are involved in differentiation into hepatocytes. However, oval cell–derived hepatocytes were DPPIV(−) and were indistinguishable from existing DPPIV(−) hepatocytes; thus, their true contribution to hepatocyte regeneration could not be determined in this model.

To further determine how significant the oval cell contribution was to hepatocyte regeneration after AAF/PH injury, we performed transplantation experiments using DPPIV(+) oval cells combined with AAF/PH injury (Fig. 3d). This transplantation model has been previously employed to trace the fate of oval cells in vivo [27, 29]. Enriched oval cells populations containing 40%–50% CK19(+) cells (2 × 10^6^/mL) were intraportally transplanted into DPPIV-deficient rats. Seven weeks following AAF/PH injury (8 weeks following transplantation), small DPPIV(+) hepatocyte clusters were observed to occupy 3.3% ± 1.3% (1%–5%) of recipient DPPIV(−) livers (*n* = 6 rats). These clusters were completely integrated into the hepatic plates and histologically identical to the surrounding DPPIV(−) hepatocytes (Fig. 3d).

These findings indicate that although oval cells did give rise to hepatocytes, they are not the primary contributor to parenchymal regeneration after AAF/PH injury.

### Sustained inhibition of hepatocyte proliferation results in extensive oval cell expansion in an undifferentiated state but no hepatocyte regeneration

**Fig. 4:**
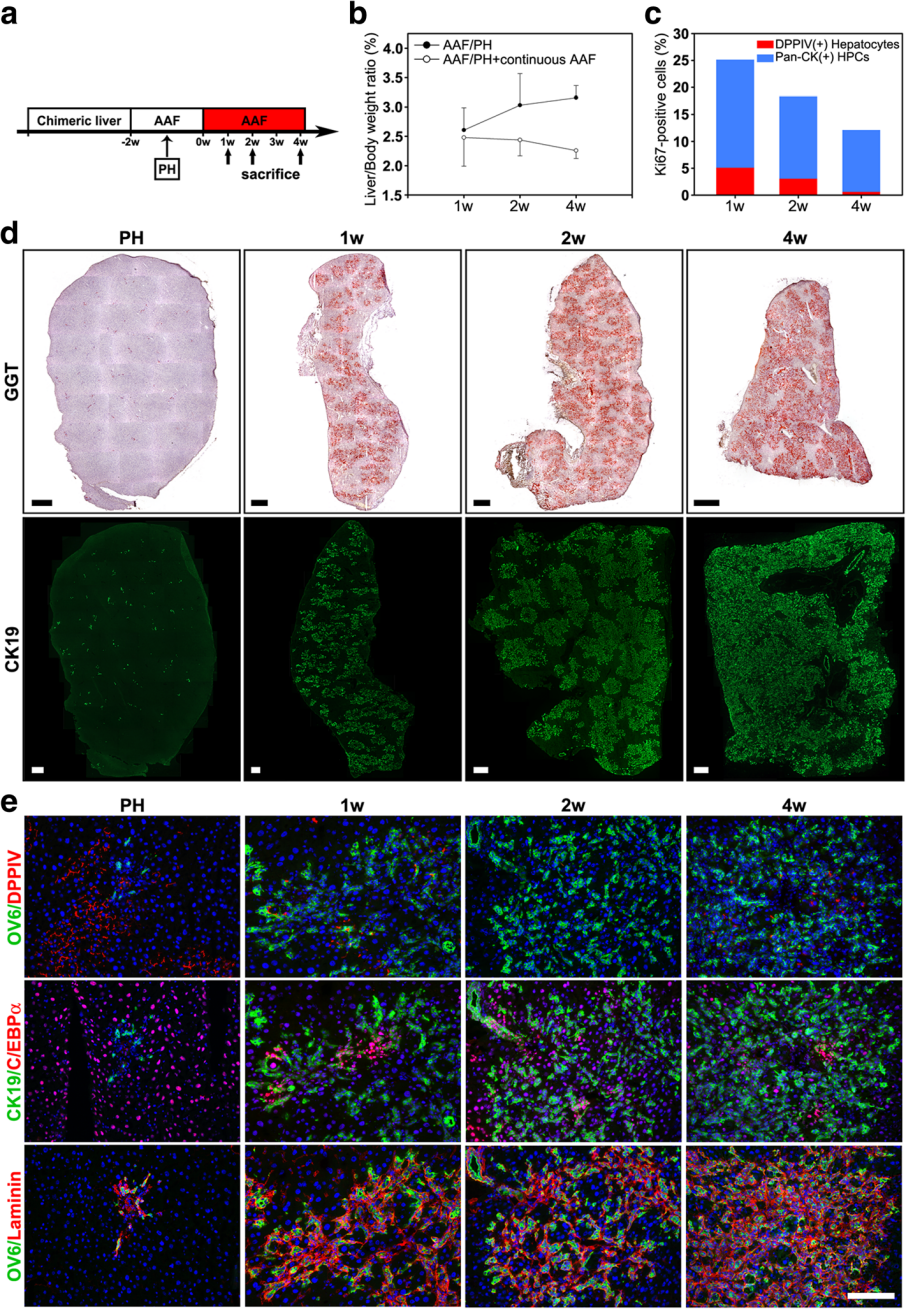
Sustained inhibition of hepatocyte proliferation resulted in more extensive oval cell expansion in an undifferentiated state but no hepatocyte differentiation. **a** DPPIV-chimeric lineage tracing system subjected to AAF/PH treatment followed by continuous AAF gavage daily for an additional 4 weeks (AAF/PH/AAF). **b** Mean liver/body weight ratios at 1, 2, and 4 weeks in AAF/PH and AAF/PH/AAF injuries. The liver/body weight ratios remained low through 4 weeks in AAF/PH/AAF injury, indicating impaired liver regeneration in this model. *P < 0.05, t*-*test, AAF/PH vs. AAF/PH/AAF at 4 weeks. **c** Quantification of proliferative activities of DPPIV(+) hepatocytes and oval cells at 1, 2, and 4 weeks in AAF/PH/AAF injury. **d** Representative images stained histochemically for GGT and with immunofluorescence for CK19 from different rats, demonstrating that continuous AAF treatment promoted a more extensive oval cell expansion with time. **e** Representative double-immunofluorescence images for DPPIV/OV6, CK19/C/EBPα, and OV6/laminin in serial liver sections at 1, 2, and 4 weeks demonstrate that oval cell proliferation diffusely infiltrated the liver parenchyma, was surrounded by thick laminin basements, and continued to express only the biliary/oval cell markers, OV6, CK19, and C/EBPα at 2 and 4 weeks. Original magnification: **d** 100×; **e** 200×. Scale bars: **d** 300 μm; **e** 100 μm

The inhibition of hepatocyte proliferation by AAF is reversible [8, 9]. After cessation of AAF, hepatocytes are released from mitotic arrest, and they can resume proliferation. We speculated that the limited contribution of oval cells to hepatocyte regeneration after AAF/PH is due to continued hepatocyte proliferation. To test whether sustained inhibition of hepatocyte proliferation would allow oval cells to regenerate hepatic parenchyma, we subjected the rats with DPPIV-chimeric livers to the AAF/PH protocol followed by continuous AAF gavage for an additional 4 weeks (AAF/PH/AAF injury) (Fig. 4a). Continuous AAF treatment resulted in high mortality after 2 weeks. We found that the livers remained small and the liver/body weight ratio remained low throughout the study periods [2.5% ± 0.5% (*n* = 6), 2.4% ± 0.3% (*n* = 6), and 2.3% ± 0.1% (*n* = 3), at 1, 2, and 4 weeks, respectively] (Fig. 4b), indicating impaired liver regeneration in this model. The livers were examined for hepatocyte differentiation of oval cells. We observed that with continuous AAF treatment, the oval cell response was more extensive and that the cells infiltrated the entire liver parenchyma (Fig. 4d). Dual immunofluorescence staining in serial sections revealed that all the oval cell proliferations were still surrounded by a thick laminin basement and that they continued to express only the biliary/oval cell markers—CK19, OV6, and C/EBPα—at 1, 2, and 4 weeks (Fig. 4e). No GGT(+)/DDPIV(−) foci containing oval cell–derived hepatocytes were found. The percentage of DPPIV(+) hepatocytes was greatly reduced (1.8% ± 0.5%; *n* = 3 rats). Ki67 expression was observed primarily in oval cells and to a limited extent in hepatocytes. The proliferative index of oval cells was 3.9-, 4.9-, and 19.2-fold higher than that of hepatocytes at 1, 2, and 4 weeks respectively (Fig. 4c). The total number of Ki67 labeling cells decreased with time. These data indicate that sustained inhibition of hepatocyte proliferation results in robust oval cell expansion in an undifferentiated state rather than in hepatocyte regeneration. Our findings suggest that continuous AAF treatment might inhibit the proliferation of new oval cell–derived hepatocytes or even kill these cells.

### Oval cells contribute substantially to hepatocyte regeneration in a noncompetitive environment

**Fig. 5:**
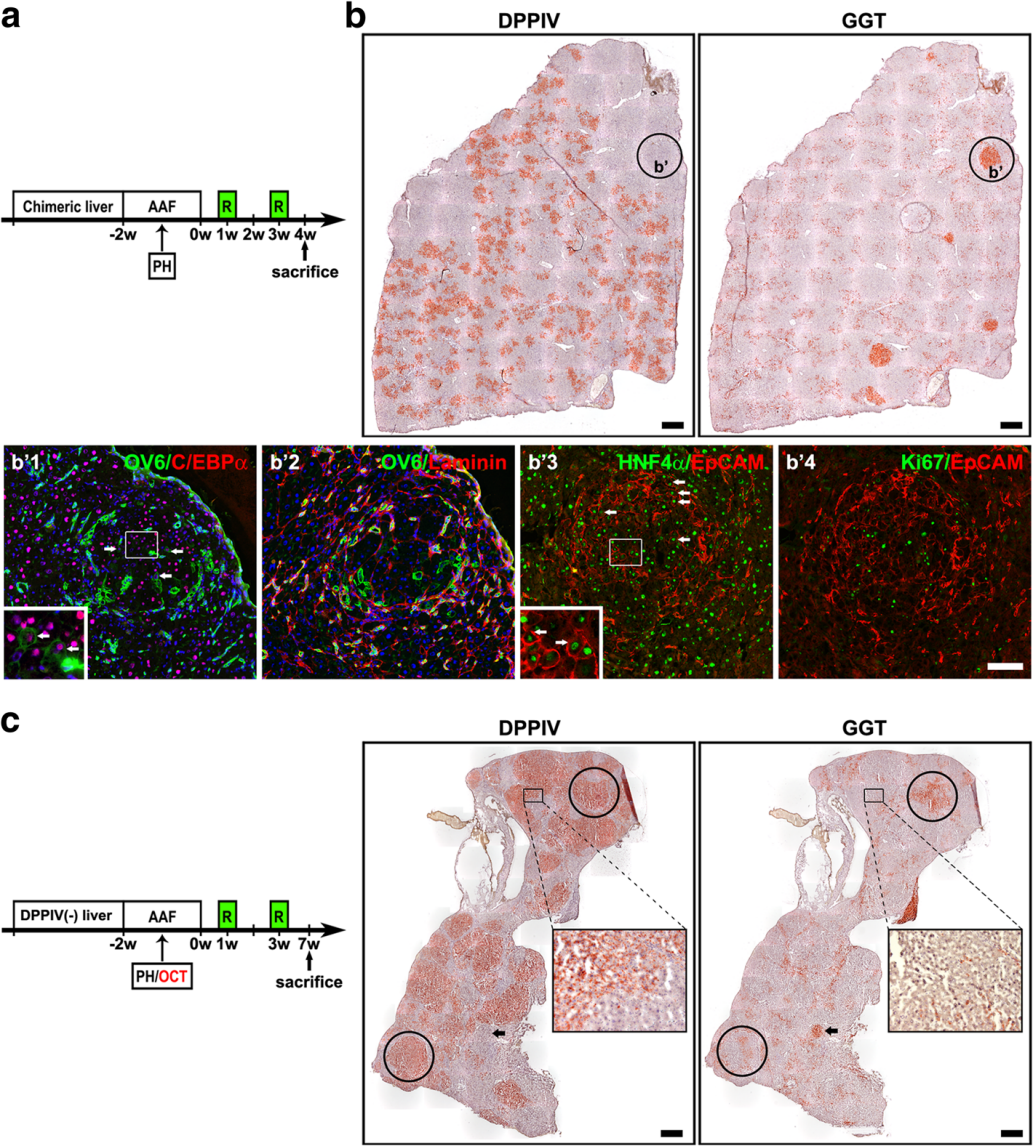
Oval cells regenerate large-scale hepatocytes in a noncompetitive environment. **a** DPPIV-chimeric lineage tracing system subjected to AAF/PH treatment followed by two doses of retrorsine 2 weeks apart at 1 and 3 weeks after cessation of AAF (AAF/PH/retrorsine). **b** A whole liver section of a DPPIV-chimeric liver at 1 week after AAF/PH/retrorsine treatment was stained for DPPIV and GGT. Oval cell proliferation infiltrated less extensively into the hepatic lobules. GGT(+)/DDPIV(−) foci of variable sizes were easily observed. These GGT(+)/DPPIV(−) foci were comprised of differentiating oval cells and newly formed hepatocytes [OV6(+)/C/EBPα(+), HNF4α(+)/EpCAM(+)] . **c** DPPIV-deficient rats received DPPIV(+) oval cells transplantation (OCT) combined with AAF/PH/retrorsine injury. After 4 weeks following AAF/PH/retrorsine injury, DPPIV(+) oval cells regenerated large-scale DPPIV(+) hepatocyte clusters. At higher magnification, DPPIV(+) oval cell–derived hepatocytes were histologically identical to the surrounding DPPIV(−) hepatocytes. Both DPPIV(+)/GGT(+) (circles) and DPPIV(−)/GGT(+) (arrow) clusters, indicating transplanted and host oval cell–derived hepatocytes respectively, were seen. Original magnification: **b** 100×/zoom magnification 200×; **c** 100×/zoom magnification 200×. Scale bars: **b** 300 μm; **b** b’1–b’4 100 μm; **c** 300 μm

To ascertain our unexpected finding using another protocol, we subjected the rats with DPPIV-chimeric livers to the AAF/PH protocol, followed by two additional doses of retrorsine (at 1 and 3 weeks after AAF/PH injury, AAF/PH/retrorsine); we then harvested the livers at 4 weeks (1 week after the 2nd dose of retrorsine, *n* = 6 rats; Fig. 5a). In contrast to AAF, retrorsine permanently blocks proliferation of hepatocytes [36]. The hypothesis in this protocol was that retrorsine irreversibly inhibits proliferation of existing hepatocytes but does not affect the new oval cell–derived hepatocytes appearing after retrorsine treatment. Our results revealed that the liver/body weight ratio was 3.0% ± 0.3%, a level in between those of the AAF/PH and AAF/PH/AAF injury models at 4 weeks. The average change in percentage of DPPIV(+) hepatocytes within the animals was − 10.7% ± 2.2% [32.1% ± 4.1% (4 weeks) vs. 42.8% ± 5.9% (PH)]. The oval cell proliferation did not infiltrate the hepatic lobules to the same extent seen in the AAF/PH/AAF model (Fig. 5b). GGT(+)/DDPIV(−) foci of variable sizes were observed adjacent to the proliferating oval cells. Dual immunofluorescence staining in serial sections confirmed that these GGT(+)/DPPIV(−) foci were composed of differentiating oval cells and newly formed hepatocytes [HNF4α(+)/EpCAM(+)] (Fig. 5b and b’1–b’4). Ki67 expression was observed in oval cells and adjacent DPPIV(−) hepatocytes clusters, indicating that they are new oval cell–derived hepatocytes; however, this expression was not observed in DPPIV(+) hepatocytes. These results suggest that oval cells give rise to hepatocytes and that the oval cell–derived new hepatocytes are responsible for parenchymal regeneration in this AAF/PH/retrorsine model.

To confirm the liver regeneration potency of oval cells, we performed DPPIV(+) oval cell transplantation experiments (2 × 10^6^/mL enriched CK19(+) oval cells populations) in the AAF/PH/retrorsine model (Fig. 5c). After 7 weeks following AAF/PH injury (4 weeks after the 2nd dose of retrorsine and 8 weeks following transplantation), large DPPIV(+) hepatocyte clusters were observed to occupy 35.4% ± 22.5% (10%–63%) of transplanted DPPIV(−) livers (*n* = 6 rats). These clusters were completely integrated into the hepatic plates and histologically identical to the surrounding DPPIV(−) hepatocytes (Fig. 5c). DPPIV(+)/GGT(+) clusters were seen, indicating that the DPPIV(+) hepatocytes were derived from the transplanted oval cells. These findings indicate that oval cells can regenerate a large-scale hepatocyte parenchyma in a noncompetitive environment.

## Discussion

In vivo lineage tracing and cell transplantation are two gold standard strategies used to assess the fate and contribution of cell types in organ regeneration [6]. In the present study, by employing lineage tracing of DPPIV(+) hepatocytes in a DPPIV-chimeric liver model and tracking the fate of DPPIV(+) oval cells in transplantation experiments in DPPIV-deficient rats, we demonstrated that hepatocyte self-replication provides the majority of hepatocyte regeneration with supplementary contribution from oval cells in rats under AAF/PH injury. By employing two models of sustained inhibition of hepatocyte proliferation following AAF/PH injury, we demonstrated that the fate of oval cells is dependent on the injury context, either expanding in an undifferentiated status or significantly regenerating the hepatocytes.

AAF/PH injury is the prototype of an oval cell activation injury model in rats. That liver regeneration in this model was entirely mediated by oval cells based on pulse-chase experiments is a long-held belief [8, 9]. Because of the lack of lineage tracing tools in rats, this concept was yet to be conclusively proven. DPPIV-chimeric livers where the DPPIV(−) hepatocytes are incapable of proliferation provide a reliable lineage tracing system to trace the respective contribution of DPPIV(−) oval cells and DPPIV(+) hepatocytes in liver regeneration in rats [25–27]. Employing chimeric experiments, we demonstrated that DPPIV(+) hepatocytes extensively proliferated after cessation of AAF to replenish the liver mass, and we clarified that hepatocytes are the primary source of hepatocyte regeneration in AAF/PH injury.

However, we observed in the AAF/PH injury that oval cells differentiated into hepatocytes after cessation of AAF treatment when hepatocytes underwent extensive proliferation. Consistent with our results, other studies in mouse oval cell–injury models have also observed that oval cells can differentiate into hepatocytes upon recovery [18–24]. By contrast, oval cells extensively expanded in an undifferentiated state rather than undergoing hepatocyte differentiation upon sustained inhibition of hepatocyte proliferation by continuous AAF treatment in the AAF/PH/AAF injury model. Upon continuing AAF administration, new oval cell–derived hepatocytes metabolized AAF and formed AAF–DNA adducts; they were thus unable to proliferate as hepatocytes. Our findings provide evidence against the hypothesis that oval cells can undergo hepatocyte differentiation in the face of severe liver injury or hepatocyte replication failure [2–5]. Similarly, in humans, the extent of the HPC reaction directly correlates with the severity of chronic liver disease and is associated with a mostly unfavorable outcome [11, 37]. In mouse models, three long-term liver injuries were all reported to result in drastic oval cell expansion and no hepatocyte generation by oval cells [23]. In these incidences of human and rodent severe chronic liver injuries and subsequent hepatocyte replication failure, the lack of HPC/oval cell–derived hepatocytes is probably because when transforming into hepatocytes, the cells acquire drug-metabolizing enzymes or express viral receptors, become susceptible to hepatotoxic agents or hepatotropic viruses, and are consequently killed [18, 38]. Robust oval cell expansion may be an adaptive but failed attempt to regenerate the severely injured liver [18].

In contrast to AAF/PH/AAF injury, we observed that oval cells can significantly regenerate hepatocytes in AAF/PH/retrorsine injury. Retrorsine is metabolized by hepatocytes through cytochrome 450 enzymes to dienic pyrroles, which form persistent adducts with protein, RNA, and DNA, generating permanent mito-inhibition and chronic injury [36, 39]. Although both injury models induced complete inhibition of hepatocyte proliferation, the two injury models differed significantly in their effect on the new oval cell–derived hepatocytes, where any new oval cell–derived hepatocytes were inhibited to proliferate or killed by continuous AAF administration in the AAF/PH/AAF injury. However, the new oval cell–derived hepatocytes emerging after retrorsine administration were not affected by the retrorsine in the AAF/PH/retrorsine injury and became the sole cells responsible for hepatocyte regeneration. Similarly, in mouse and zebrafish studies, selective inhibition of hepatocyte proliferation by β1-integrin knockdown or p21 overexpression in mice and extreme deletion of hepatocytes by metronidazole in zebrafish has been reported to lead to hepatocyte regeneration by oval cells [14–17]. The findings of these studies coupled with that of ours suggest that oval cells can significantly regenerate hepatocytes in a chronic selective injury and/or noncompetitive environment.

**Fig. 6:**
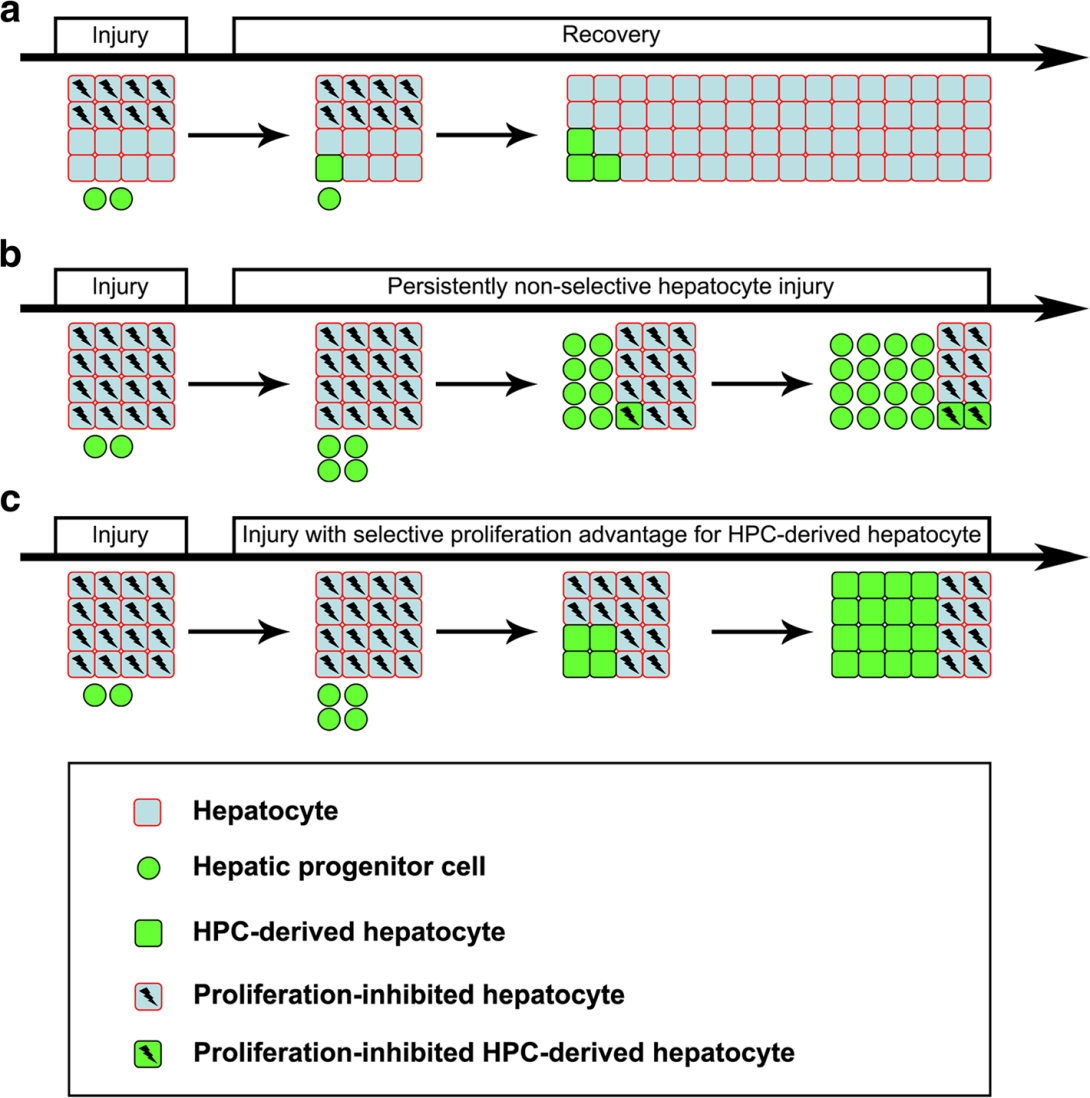
Schematic of cellular mechanisms governing liver regeneration after injuries. Hepatocytes and HPCs are called upon to regenerate the tissue loss in virtually all forms of liver injuries. **a** When the liver injury is mild to moderate or short term, surviving hepatocytes quickly proliferate and HPCs undergo hepatocyte differentiation. Surviving hepatocytes generally outnumber HPC-derived hepatocytes (approximately 30–100 to 1) and are the primary cells to regenerate hepatocytes. **b** Upon continuous and chronic liver injuries, hepatocytes and HPC-derived hepatocytes are equally and continuously eliminated. HPCs undergo extensive proliferation in an attempt to compensate for the tissue loss. **c** When native hepatocytes are extensively ablated or selectively inhibited to proliferate, HPCs can significantly regenerate hepatocytes over time

On the basis of the results of our study as well as other major studies [10–24], we summarized liver regeneration following various injuries into three types (Fig. 6). Hepatocytes and HPCs/oval cells are called upon to regenerate the tissue loss in virtually all forms of liver injuries. First, when the liver injury is mild-to-moderate or short-term [18–24], surviving hepatocytes quickly proliferate, and HPCs/oval cells undergo hepatocyte differentiation. Typically, surviving hepatocytes outnumber HPC/oval cell–derived hepatocytes (approximately 30–100 to 1) and are the primary cells responsible for regenerating hepatocytes [5]. Second, upon continuous and chronic liver injuries, hepatocytes and HPC/oval cell–derived hepatocytes are equally and continuously eliminated. HPCs/oval cells undergo extensive proliferation in an attempt to compensate for the tissue loss [18, 19, 23]. Third, in a context where native hepatocytes are extensively ablated or selectively inhibited to proliferate, HPCs/oval cells can significantly regenerate hepatocytes [14–17]. These findings are comparable with those obtained in cell transplantation in the liver or hematopoietic system [40], where selective inhibition or extensive ablation is required for hepatocyte or bone marrow replacement by transplanted hepatic cells or hematopoietic stem cells. Our results help reconcile the discrepancies among studies defining sources of liver regeneration.

From an evolutionary perspective, the liver must have developed multiple mechanisms of repair, and it can thus call upon more than one cell type to support liver regeneration when confronted with diverse toxic insults [1–3]. Among such cells, hepatocytes are the most efficient cell type for repair. Other cell types may serve as a back-up system to hepatocyte differentiation in order to ensure the maintenance of liver integrity. Paradoxically, after becoming hepatocytes, the cells are susceptible to diverse hepatotoxic agents or hepatotropic viruses. Ultimately, removal of the injurious agents or viruses is the key to promoting liver repair and saving lives [3].

## Conclusions

The present study demonstrated that hepatocytes are the primary cells responsible for the regeneration of liver mass in rats under AAF/PH injury. Oval cells can significantly regenerate hepatocytes in a noncompetitive environment. Oval cells maintain in an undifferentiated state and fail to regenerate liver mass upon continuously nonselective liver injury. These findings suggest that removal of the injurious agents or viruses is the key to promoting liver repair.

## Abbreviations

AAF/PH: 2-acetylaminofluorene/partial hepatectomy
C/EBPα: CCAAT enhancer binding protein alpha
CPS1: Carbamoyl-phosphate-synthetase 1
DPPIV: Dipeptidyl peptidase IV
EpCAM: Epithelial cell adhesion molecule
GGT: Gamma-glutamyl-transpeptidase
HNF4α: Hepatocyte nuclear factor 4α
HPCs: Hepatic progenitor cells
